# 3D Carbon Microelectrodes with Bio-Functionalized Graphene for Electrochemical Biosensing

**DOI:** 10.3390/bios8030070

**Published:** 2018-07-19

**Authors:** Suhith Hemanth, Arnab Halder, Claudia Caviglia, Qijin Chi, Stephan Sylvest Keller

**Affiliations:** 1 Department of Micro- and Nanotechnology, Technical University of Denmark, 2800 Kgs. Lyngby, Denmark; suhem@nanotech.dtu.dk (S.H.); clauc@nanotech.dtu.dk (C.C.); 2 Department of Chemistry, Technical University of Denmark, 2800 Kgs. Lyngby, Denmark; arhal@nanotech.dtu.dk (A.H.); cq@kemi.dtu.dk (Q.C.)

**Keywords:** 3D carbon microelectrodes, graphene oxide, glucose, electrochemical biosensor

## Abstract

An enzyme-based electrochemical biosensor has been developed with 3D pyrolytic carbon microelectrodes that have been coated with bio-functionalized reduced graphene oxide (RGO). The 3D carbon working electrode was microfabricated using the pyrolysis of photoresist precursor structures, which were subsequently functionalized with graphene oxide and enzymes. Glucose detection was used to compare the sensor performance achieved with the 3D carbon microelectrodes (3DCMEs) to the 2D electrode configuration. The 3DCMEs provided an approximately two-fold higher sensitivity of 23.56 µA·mM^−1^·cm^−2^ compared to 10.19 µA mM^−1^·cm^−2^ for 2D carbon in glucose detection using cyclic voltammetry (CV). In amperometric measurements, the sensitivity was more than 4 times higher with 0.39 µA·mM^−1^·cm^−2^ for 3D electrodes and 0.09 µA·mM^−1^·cm^−2^ for the 2D configuration. The stability analysis of the enzymes on the 3D carbon showed reproducible results over 7 days. The selectivity of the electrode was evaluated with solutions of glucose, uric acid, cholesterol and ascorbic acid, which showed a significantly higher response for glucose.

## 1. Introduction

Electrochemical biosensors are one of the most successful sensor technologies in terms of real-world applications and commercial maturity [1,2,3,4,5]. Therefore, the research and development of electrochemical biosensors is currently one of the most active areas for various important applications, such as diagnostics [6], environmental monitoring [7], food quality control [8], security and defense [9]. Electrodes are a central part for all electrochemical applications and are used as the transducer in electrochemical biosensing devices [10]. Therefore, the constant effort to improve electrodes has played an integral role in encouraging the advancement of electrochemical biosensors. Various conductive substrates have been used for the development of electrodes in the past few decades [11]. Among them, carbon-based electrodes are the most popular ones. Carbon-based materials can be found as many different allotropes, such as graphite, glassy carbon, carbon fibers, diamond, fullerene and carbon nanotubes (CNTs) [12]. Due to their wide availability, low-cost, high stability, high conductivity and excellent electrochemical performance, these carbon-based materials have become an obvious choice for a wide range of electrochemical applications, such as biosensors, supercapacitors or fuel cells [11]. Furthermore, the slow kinetics for the oxidation of carbon facilitate its use in wide potential ranges (anodic direction) for various electrochemical methods, which provides a crucial advantage compared to various metal-based electrodes [13]. However, the typically used carbon-based electrodes provide a two-dimensional (2D) surface area. In electrochemical sensing, the amplitude of the recorded signals is dependent on the surface area of the electrode. Therefore, a rational design of the electrodes and an increase in electrode surface area for the same overall electrode footprint area are potential strategies for improving the performance of electrochemical biosensors. A frequently used approach to achieve this involves the integration of nanomaterials, such as CNTs or nanoparticles, into the sensor electrode [14] Several microfabrication processes have been reported for the fabrication of three-dimensional (3D) microelectrodes [15,16,17,18,19]. In the last few years, 3D electrodes have shown very promising results for diverse electrochemical applications [20,21]. However, these electrodes are frequently still lacking consistency in their performance due to poor control of the 3D structural definition.

In this work, microfabricated 3D carbon electrodes are evaluated for electrochemical biosensing and their biosensor performance is compared with the one of 2D carbon electrodes fabricated with a similar approach. For our study, one of the simplest and most cost efficient techniques, Carbon Microelectromechanical systems (C-MEMS) technology, is used to fabricate 3D pyrolytic carbon microelectrodes (3DCMEs). In C-MEMS, a patterned polymer precursor template is pyrolyzed at high temperatures (≥900 °C) to obtain well-defined 3DCMEs [22,23]. Similar to other carbon materials, pyrolytic carbon has several attractive properties, such as a wide electrochemical potential window, ease of surface functionalization and biocompatibility [11,24,25,26]. Furthermore, the pyrolytic carbon electrodes are coated with chemically synthesized reduced graphene oxide (RGO), which has been functionalized by polyethyleneimine (PEI) to provide stable accommodation of enzymes for biosensing and additionally increase the surface area of the electrode. 

Graphene is one of the most studied nanomaterials during the last decade due to its extraordinary properties [27]. Although pristine graphene itself is a zero band gap material [28], the suitable functionalization of chemically synthesized graphene can convert it into an ideal material for many sensing applications [29]. The RGO is bio-functionalized with the enzyme glucose oxidase (GOx) and glucose measurements are performed as an example for an application of the 3DCMEs in enzyme-based electrochemical biosensing. The stability and selectivity of the biosensor with 3D electrodes are studied. Throughout the study, the performance of 3D electrodes in biosensing is compared with 2D electrodes. Finally, glucose level measurements in real human blood serum samples are demonstrated using the 3DCMEs. 

## 2. Materials and Methods

### 2.1. Microfabrication of 3D Carbon Microelectrodes 

**Figure 1:**
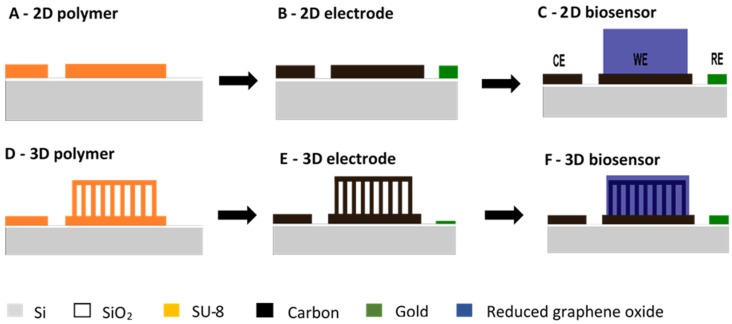
Schematic of 2D (**A**–**C**) and 3D (**D**–**F**) carbon biosensor fabrication: (**A**,**D**) polymer precursor template fabricated by multiple UV photolithography processes with the negative epoxy photoresist SU-8; (**B**,**E**) pyrolytic carbon fabricated by pyrolysis of the corresponding polymer precursor template; (**C**,**F**) functionalization of WE with reduced graphene oxide.

The design and fabrication of the microelectrodes have been reported previously [30]. The microelectrode chips had overall dimensions of 1 cm × 3 cm. The integrated three electrode configuration consisted of a pyrolytic carbon working (WE), counter electrode (CE) and an Au pseudo reference electrode (RE). The circular working electrode had a diameter of 4 mm. For the electrode fabrication, a 600-nm thick SiO_2_ layer was deposited on 4-inch Si wafers by thermal oxidation. A first step of UV photolithography with a 17-µm thick layer of the negative epoxy-based photoresist SU-8 2035 (Microchem, Westborough, MA, USA) was performed to define the pattern of the 2D working (WE) and counter electrode (CE) (Figure 1A). For the fabrication of the 3D electrode, an additional 98-µm thick film of SU-8 2075 was spin-coated on the wafer. Subsequently, the SU-8 was exposed with 147 mJ·cm^−2^ to define supporting micropillars. With partial exposure, we used a dose of 28 mJ·cm^−2^ to pattern the suspended layer, which was followed by a development step (Figure 1D). The SU-8 precursor templates were pyrolyzed at 900 °C for 1 h in N_2_ atmosphere to obtain the 2D (Figure 1B) and 3D (Figure 1E) carbon microelectrodes. A pseudo gold (Au) reference electrode (RE) and contact leads with a thickness of 200 nm were deposited through a shadow mask using e-beam evaporation. After pyrolysis, a 6-µm thick film of SU-8 was spin-coated and patterned to partially passivate the contact leads. The open part of the contact leads was used for the connection of the electrodes to the potentiostat.

### 2.2. Reduced Graphene Oxide (RGO) Synthesis

The bio-functionalized graphene was synthesized according to a method that was reported previously in reference [31]. Graphene oxide was used as a starting material and was synthesized by the modified Hummers method [32]. In the next step, pre-synthesized graphene oxide was reduced and covalently functionalized by branched polymer polyethylenimine (PEI) in a one-step reaction method [33]. After this, the as-synthesized RGO-PEI was modified with ferrocene carboxylic acid to achieve the redox activity of the biosensing material. The final redox active modified RGO-PEI-based material was combined with biosensing elements (enzyme) and directly deposited on the electrode surface.

### 2.3. Biosensor Preparation

The ferrocene functionalized RGO-PEI material was dispersed in 1 mL of 0.5% ethanolic Nafion^®^ solution by sonication. The electrode chips were pre-cleaned in O_2_ plasma for 3 min. The solution was drop casted on the WE area of the 2D (Figure 1C) and 3D (Figure 1F) carbon microelectrode chip, before being dried at room temperature overnight. Finally, for the preparation of the glucose biosensing electrode, 10 μL of glucose oxidase (GOx) solution (10 mg/mL) in a phosphate buffer (PBS, 10 mM, pH of 7.0) were drop casted on the WE surface. The bio-functionalized 3D electrodes with enzymes were dried at 4 °C overnight. Before performing the electrochemical biosensing measurements, the electrodes were repeatedly washed with buffer solutions (PBS, 10 mM, pH of 7.0) to remove the loosely bound enzymes. 

### 2.4. Electrochemical Biosensing

All electrochemical measurements in this present work were obtained at room temperature (23 ± 2 °C) using an Autolab System (Metrohm Nordic ApS, Glostrup, Denmark) operated by the NOVA 1.10 software or by a CHI 760C electrochemical workstation equipped with a Faradaic cage. The electrochemical behavior of the bio-functionalized 2D/RGO-PEI/GOx and 3D/RGO-PEI/GOx electrodes was studied by cyclic voltammetry (CV) in 50 µL of 10 mM PBS electrolyte (pH 7). The WE potential varied from −0.2 V to 0.5 V at a scan rate of 20 mVs^−1^. Glucose sensing was performed using CV in the same electrolyte with different concentrations of glucose. Chronoamperometric measurements for glucose sensing were obtained in 10 mM PBS electrolyte solution (pH 7) with the working electrode potential fixed at 0.15 V and 0.1 V for the 2D/RGO-PEI/GOx and 3D/RGO-PEI/GOx carbon electrodes, respectively. The operational stability of the bio-functionalized graphene-based 3D/RGO-PEI/GOx electrodes was tested. For this purpose, amperometric measurements were performed every day for seven days using the 3D electrodes stored at 4 °C. The selectivity of the biosensor was evaluated in the presence of other coexisting common easily oxidizable analytes, such as cholesterol, ascorbic acid and uric acid. The glucose level in real human blood serum samples obtained from a local Danish hospital was measured both with the 3D/RGO-PEI/GOx carbon electrodes and a commercially available blood glucose measurement device (Aviva Accu-chek). The samples were handled according to the guidelines of the hospital. For evaluation of the glucose level, human blood serum (3–9 µL) was directly added to the electrolyte solution (50 µL, 10 mM PBS, pH 7) on the 3D electrode surface and the steady state current was measured. Three individual measurements were performed for two different samples.

## 3. Results and Discussion

**Figure 2:**
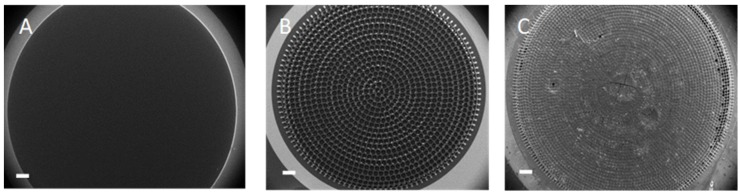
(**A**) 2D WE (**B**) 3D WE and (**C**) 3D WE with RGO-PEI/GOx (scale bar: 500 µm).

The 2D and 3D carbon microelectrodes for enzyme-based electrochemical biosensing were fabricated as reported previously in references [20,30]. Figure 2A,B shows the fabricated 2D and 3D carbon electrodes, while Figure 2C shows the 3D electrode after drop casting of the graphene oxide. The thickness of the 2D pyrolytic carbon electrodes was 2.1 µm, which is considerably lower than the initial thickness of the SU-8 films due to shrinkage during pyrolysis. Similarly, the height of the 3D pyrolytic carbon structures decreased to 44 µm. The 3D microelectrodes consisted of pillars with a diameter of 6.7 µm and pitch of 50 µm. The suspended layer on the top of the pillars had holes with a diameter of 29 µm and pitch of 50 µm. The WE diameter was 4 mm. The surface area of the bare 3DCMEs was approximately twice the surface area of the 2D electrode [30].

**Figure 3:**
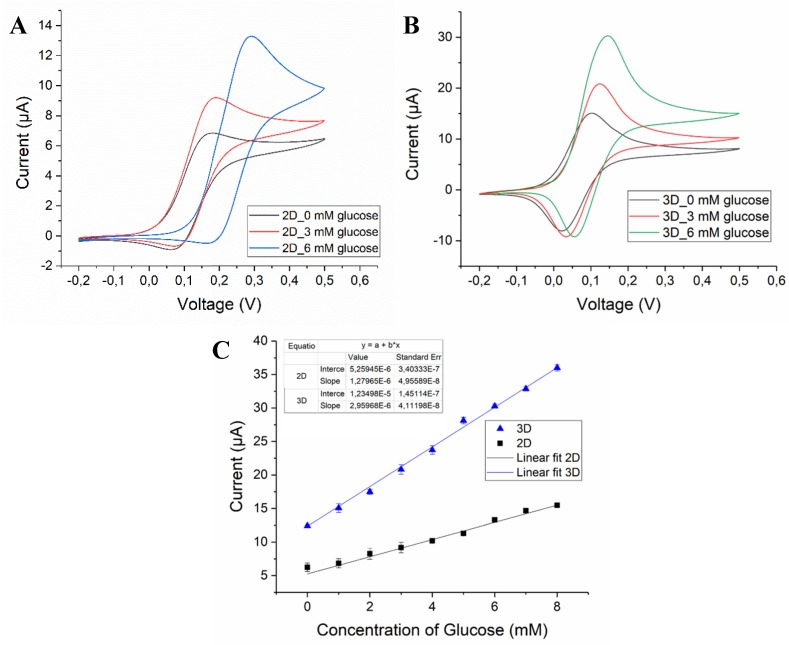
Cyclic voltammogram of (**A**) 2D/RGO-PEI/GOx and (**B**) 3D/RGO-PEI/GOx carbon electrodes in 10 mM PBS (pH 7), 3 mM glucose and 6 mM glucose at scan rate of 20 mV/s. (**C**) Corresponding calibration plot obtained from the concentration of glucose vs. current (N = 3).

The electrochemical behavior of the bio-functionalized 3D electrode and glucose biosensing was first studied by cyclic voltammetry. Figure 3A,B exhibits the cyclic voltammograms recorded with 2D and 3D pyrolytic carbon electrodes coated with RGO and functionalized with GOx (2D/RGO-PEI/GOx and 3D/RGO-PEI/GOx, respectively). The voltammetric response of the bio-functionalized 3D and 2D electrodes in 10 mM PBS buffer (0 mM glucose) remained constant over hundreds of scans in the potential window of −0.2 to 0.5 V, which clearly indicated that the electrode material combined with the graphene-based biosensing material was stable. For CVs recorded with increasing glucose concentration, the anodic peak current increased, which is a typical characteristic of electron transfer mediated bioelectrocatalysis.

As clearly seen in Figure 3, the CVs obtained with 3D/RGO-PEI/GOx electrodes in PBS buffer without glucose displayed a significantly higher current response (3-fold) compared to the 2D/RGO-PEI/GOx electrode, which holds great promise for the development of a highly sensitive biosensing platform. Similarly, the CVs recorded for 3D carbon electrodes in the solutions containing glucose displayed a higher response when compared to 2D carbon electrodes for measurements. Figure 3C shows that the extracted peak current increased linearly with increasing glucose concentrations in the physiologically relevant range. The calculated sensitivities for 2D/RGO-PEI/GOx and 3D/RGO-PEI/GOx carbon electrodes were 10.19 µA·mM^−1^·cm^−2^ and 23.56 µA·mM^−1^·cm^−2^, respectively. 

**Figure 4:**
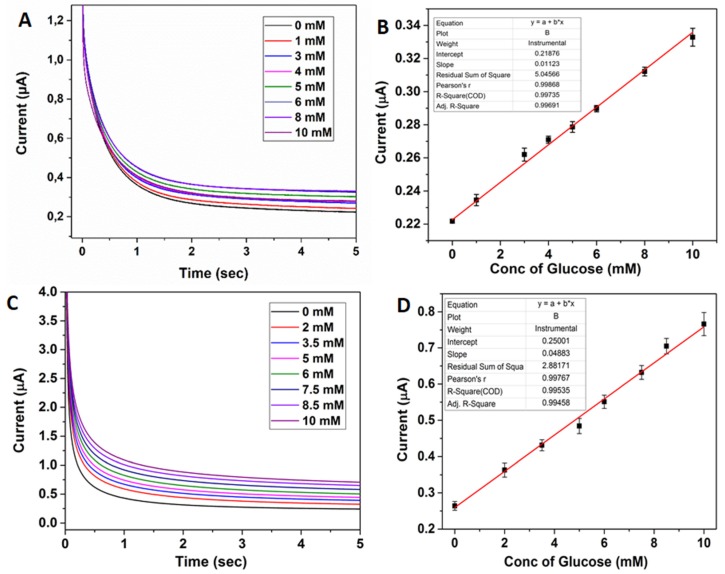
(**A**) Chronoamperometric sensing of glucose (0–10 mM) with 2D/RGO-PEI/GOx pyrolytic carbon electrodes in 10 mM PBS (pH 7) (**B**) Corresponding calibration plot obtained from concentration of glucose vs. current at 4 s. (**C**) Chronoamperometric sensing of glucose (0–10 mM) with 3D/RGO-PEI/GOx pyrolytic carbon electrodes in 10 mM PBS (pH 7) (**D**) Corresponding calibration plot obtained from concentration of glucose vs. current at 4 s.

Furthermore, chronoamperometric glucose biosensing was performed by using both the 2D/RGO-PEI/GOx and 3D/RGO-PEI/GOx electrodes (Figure 4). The amperometric measurements were obtained in 10 mM PBS electrolyte solution (pH 7) with the working electrode potential fixed at 0.15 V for 2D electrodes and 0.1 V for 3D electrodes. For both electrode configurations, a linear range was achieved until 10 mM, which overlaps well with the normal human physiological blood glucose levels (5–7 mM). The lower limit of detection (LOD) for 3D/RGO-PEI/GOx was 1.2 µM and for 2D/RGO-PEI/GOx electrodes was 3 µM. Furthermore, the calculated sensitivity for chronoamperometric glucose biosensing was 0.39 µA·mM^−1^·cm^−2^ and 0.09 µA·mM^−1^·cm^−2^ for 3D/RGO-PEI/GOx and 2D/RGO-PEI/GOx electrodes, respectively. This means that the LOD in amperometric glucose sensing is 2.5 times and the sensitivity is around 4.3 times higher for the 3D electrode due to the larger active surface area.

**Figure 5:**
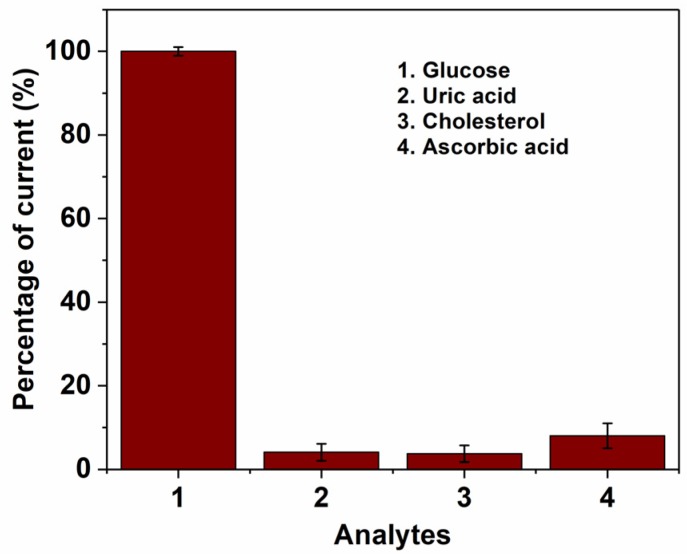
Influence of common electroactive interferences with (1) glucose (2 mM), (2) Uric acid (5 mM), (3) Cholesterol (5 mM) and (4) Ascorbic acid (5 mM) measured with 3D/RGO-PEI/GOx pyrolytic carbon electrodes. Working potential = 0.15 V, (n = 3).

In order to evaluate a realistic application of the 3DCMEs coated with RGO, the selectivity of the sensor towards some common electroactive species existing in human blood samples, such as cholesterol, ascorbic acid and uric acid, was studied (Figure 5). As seen from the amperometric measurement with GOx, no significant interferences from these compounds were observed. Furthermore, the negatively charged polymer (Nafion^®^) improves selectivity by repelling the uric acid and ascorbic acid through electrostatic repulsion.

**Figure 6:**
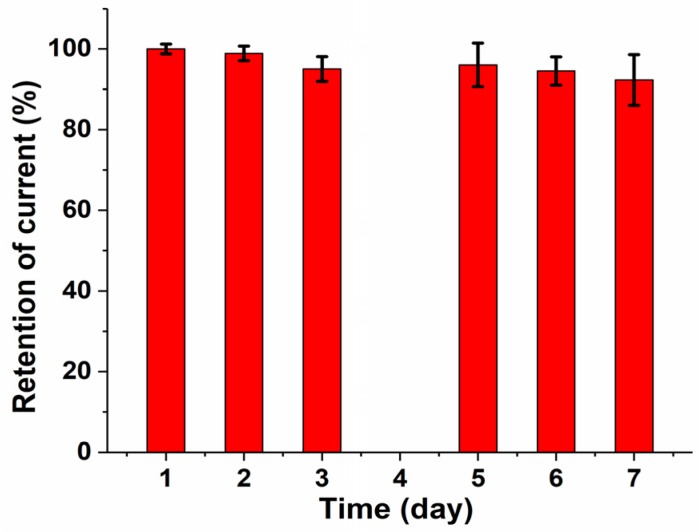
Response stability of 3D/RGO-PEI/GOx carbon electrodes stored for 7 days at 4 °C.

The stability of the bio-functionalized graphene-based 3D electrodes during storage was tested. Figure 6 shows that a decrease of 5% in the biosensor response was observed after 3 days and a decrease of 8% after 7 days. The slight change in biosensor response was attributed to the limited stability of the immobilized enzymes.

The 3D/RGO-PEI/GOx carbon electrodes were further tested as sensors to evaluate the glucose level in real human blood serum samples. The obtained results were comparable with data from the commercially available self-monitoring blood glucose device for the same samples (Table 1). The relative standard deviation (RSD) values for the measurements were found to be between 2% and 5%. The results confirmed that the fabricated 3D electrodes functionalized with RGO can be used for glucose level measurements in real human blood samples without any prior sample pre-treatment.

## 4. Conclusions

An enzyme-based electrochemical biosensor was developed with bio-functionalized 3D carbon as the working electrode. The 3D/RGO-PEI/GOx pyrolytic carbon electrodes provide a higher sensitivity (0.39 µA mM^−1^·cm^−2^) and lower limit of detection (1.2 µM) for amperometric glucose sensing compared to the 2D/RGO-PEI/GOx electrodes. The lower limit of detection and sensitivity of 3D electrodes were 2.5 times and 4.3 times higher than that of the 2D electrodes, respectively. The developed enzymatic biosensor was highly selective and stable over time for glucose sensing. The electrodes were successfully applied for glucose detection in real blood serum. With the more sensitive 3D carbon microelectrodes, the developed sensor platform could be ideal for highly sensitive electrochemical biosensors.

## Figures and Tables

**Table 1:** Amperometric measurements of glucose in real blood serum samples (n = 3).

Blood Sample	Glucose Concentration Measured with 3D Carbon Electrodes	Glucose Concentration Measured with Commercial Device	RSD
Sample 1	4.5 mM	4.8 mM	3.1%
Sample 2	5.9 mM	6.1 mM	4.7%
